# Development and Validation of a Parent-Based Program for Preventing Gaming Disorder: The Game Over Intervention

**DOI:** 10.3390/ijerph16111984

**Published:** 2019-06-04

**Authors:** Angel Yee-lam Li, Chor-lam Chau, Cecilia Cheng

**Affiliations:** 1 Department of Psychology, The University of Hong Kong, Pokfulam, Hong Kong, China; lylargle@gmail.com; 2 Department of Psychology, University College London, Bloomsbury, London WC1E 6BT, UK; sam.chau.15@ucl.ac.uk

**Keywords:** addiction, compulsive gaming, intervention, pathological gaming, problematic gaming, prevention, video game

## Abstract

Since the inclusion of gaming disorder in the fifth edition of the Diagnostic and Statistical Manual of Mental Disorders (DSM-5) as a condition for further study, there has been an increasing consensus that problematic gaming can be detrimental to mental health, yet efforts in preventing such problems from emerging have been limited. To address this gap, we developed the Game Over Intervention (GOI), a parent-based program designed based on the frameworks of ecological systems theory and self-determination theory. This study aimed to test the efficacy of the new program using the method of a randomized controlled trial, with the control condition being a program for effective learning. Participants were the parents of upper primary school students, with 163 (77% women; *M*_age_ = 42.70) and 199 (83% women; *M*_age_ = 41.82) partaking in the intervention and the control conditions, respectively. Participants rated their children’s gaming time, exposure to violent video games, and symptoms of gaming disorder at three time points: baseline, one week after intervention, and three months after intervention. The results indicate a general reduction in these three criteria across the three-month period. Our study provides tentative evidence demonstrating the effectiveness of the GOI in mitigating some gaming-related problems.

## 1. Introduction

Video gaming is a favourite pastime for many boys and girls. A survey estimated that as high as 91% of children and adolescents aged between 2 and 17 years play video games [1]. Although video gaming can bring some socialization benefits [2,3], compulsive or addictive game play is a widespread concern among parents, teachers, policymakers, and the mass media. Frequent gaming predisposes children to risks of gaming disorder [4,5], but few studies have examined what can be done to help children gain better control over their gaming behaviour and prevent them from gaming addiction. The overarching objective of this study is to address this gap by designing a parent-based intervention, namely, the Game Over Intervention (GOI), and to evaluate it using a randomized controlled trial.

Apart from gaming disorder, children’s exposure to violent video games is another issue of concern. A recent study revealed that over 30% of primary school students play violent video games weekly [6]. Given that systematic reviews have provided evidence that violent video game exposure is positively associated with aggression [7] and studies have reported that both constructs are related to gaming disorder [8,9], elements targeting the reduction of violent video game exposure are included in the GOI in addition to those targeting the prevention of gaming disorder symptoms.

### 1.1. An Overview of Gaming Disorder: Diagnostic Criteria, Consequences, and Etiology

In the latest, fifth edition of the Diagnostic and Statistical Manual of Mental Disorders (DSM-5) [10], gaming disorder is listed as a condition for further study. Gaming disorder is suggested to comprise nine diagnostic criteria [11,12], for example, spending an increasing amount of time to play video games so as to sustain the same levels of satisfaction (tolerance) and gaming to get away from or alleviate unpleasant emotions (escape). Studies from the past decades have shown that gaming disorder is related to psychosocial, physical, and other daily life problems [13,14,15,16,17,18].

Acknowledging the potential harm of gaming disorder, researchers have conducted investigations to identify the etiological factors and mechanisms of the disorder. Studies have shown that gaming disorder is associated with parental and familial factors including but not limited to insufficient parental care, excessive parental control, poor parent–child relations, and family disharmony and dysfunction [19,20,21,22]. These associations may be explained by both ecological systems theory [23] and self-determination theory [24].

### 1.2. Filling the Gap in Gaming Disorder Prevention Research: The Game Over Intervention

Although there are many studies conducted to identify predictors of gaming disorder, there has been scant effort in investigating ways to change such predictors in an effort to prevent the disorder. In a sample of college students, Yie et al. designed, implemented, and evaluated a web-based intervention targeted to reduce time spent on gaming [25], which has been identified as an antecedent to gaming disorder [26]. During the intervention, participants received individualized messages about the consequences of excessive gaming and suggestions for maintaining a healthy life-style. They were also instructed to monitor the amount of time they spent playing video games using an online logging system. However, no major changes in gaming behaviour were observed as a result of the intervention. For adolescents, De Leeuw et al. modified an existing universal health education program to include Internet literacy training (e.g., teaching coping strategies for online deceptions) and tested it in a cohort of secondary school students [27]. After a year of attending weekly trainings, a large portion of students stopped playing video games, but those who continued playing reported more symptoms of gaming disorder.

To the best of our knowledge, existing preventive interventions for gaming disorder are all individual based. However, a recent systematic review indicates that gaming disorder is associated with both personal and interpersonal factors [28]. The social context, specifically the family environment, is especially influential for young children. According to the ecological systems theory [29], effective microsystems (i.e., family, school, and peer group) interact to facilitate growth and development in children. If one of these systems is not functioning optimally, it will influence other interconnected systems and result in problematic behaviour. For information technology addiction, a study revealed that, among adolescents with low levels of effortful control, parental monitoring generally serves as a protective factor against Internet addiction through reducing deviant peer affiliation [30]. These findings imply that if family functioning, particularly patterns of parent–child interactions, can be strengthened, other settings that children embedded in can also be improved, and thus help them prevent information technology addiction. On the basis of the interactive perspective of this eclectic theoretical framework, preventive effort toward mitigating gaming disorder symptoms should extend to the family level in addition to the individual level.

The GOI is a universal parent-based intervention developed to empower parents with the knowledge, attitudes, and skills to cultivate positive parenting and family environments, which serve as protective factors against gaming disorder in children. On the basis of the self-determination theory [24], positive parenting should be characterized by a capacity to promote the fulfilment of three basic psychological needs, the need for autonomy, relatedness, and competence. If children cannot fulfil their basic needs in one social environment, they may turn to other social environments to seek need gratification. Przybylski et al. concluded in their review that the video game environment contains elements that can fulfil player’s basic needs, which makes gaming intrinsically motivating [31]. If individuals’ basic needs are deprived in reality, they may engage in compulsive gaming as an attempt to compensate for their unfulfilled needs in the game world. It is possible that for children whose basic needs cannot be gratified in their family environment (e.g., authoritarian parenting, poor parent–child relations), they may have greater proclivity to engage in obsessive gaming as a form of compensation, with concurrent reduced likelihood to internalize and integrate external regulations pertaining to gaming. Over time, these children can become at risk for developing gaming disorder. 

In these respects, a developmentally eclectic intervention can more comprehensively address the ecological risk factors of gaming disorder. Hence, we propose that coevally fostering the satisfaction of basic needs in the family setting may be more effective than individual-level intervention per se. On the basis of the self-determination theory, we designed three modules in the GOI, with each module addressing a distinct basic need. The three modules comprise parental monitoring, parental care, and psychoeducation, designed to foster the fulfilment of needs for autonomy, relatedness, and competence, respectively.

### 1.3. Modules of the GOI: Parental Monitoring, Parental Care, and Psychoeducation

The first module of the GOI centres on parental monitoring, which has been found to be a protective factor for Internet and gaming addictions [32]. Through paying close attention to when, what, and with whom children game, parents can intervene as soon as symptoms of gaming disorder arise. To strengthen this protective factor, the module of parental monitoring aims to explain how parents can stay informed about their children’s gaming activities without depriving their need for autonomy. Previous studies have shown that when performed effectively, parental monitoring can be viewed by children as supportive instead of controlling [33]. Parents with the knowledge of what their children are doing, where they are at, and who they are interacting with can guide their children to better explore their potential paths of development and build a sense of autonomy in the process [34]. 

The second module revolves around parental care, another factor that has been identified as mitigating symptoms of Internet and gaming addictions [20]. We thus incorporated a module into the GOI that includes elements of parental care that can gratify relatedness needs. These elements consist of (1) expressions of warmth, (2) responsiveness to needs, and (3) cultivation of shared positive emotions [35]. 

The third module focuses on psychoeducation, a key aspect in the intervention of gaming disorder [36]. In the GOI, this module seeks to educate parents on the “ABCs” (i.e., antecedents, behaviours, and consequences) of gaming. Having a better understanding of video gaming, such as recognizing children’s gaming motives, parents can offer need-specific alternative activities and structure surrounding those activities. This should help their children develop a sense of competence [37]. Overall, the psychoeducation module is complementary to the parental monitoring and parental care modules. Through a greater knowledge of gaming, parents can provide better gaming-specific parenting to their children [38]. 

### 1.4. Overview of Study and Hypotheses

The GOI is, to our knowledge, the first universal preventive intervention for gaming disorder targeted at parents. It follows the guidelines for psychological prevention established by the American Psychological Association [39] in that the intervention program should be both theory- and evidence-based. We chose to implement the intervention in the parents of upper primary school students because it has been proposed that the earlier the intervention, the stronger the effects will be [40]. 

To evaluate the effectiveness of the GOI, the method of a randomized controlled trial was used. There were two conditions: intervention and control. In the intervention condition, participants received the GOI; in the control condition, participants received generic intervention on effective learning. In addition to assessing the effects of GOI on children’s levels of gaming disorder, we also measured its impact on their gaming frequency and exposure to violent video games, as both factors are proximal predictors of gaming disorder and have their own implications [7,9,41,42]. It is expected that both immediately and three months after partaking in the GOI, parent participants in the intervention condition would report that their children (a) spend less hours on gaming per week, (b) have lower exposure to violent video games, and (c) have fewer symptoms of gaming disorder compared with those who participated in the control condition.

## 2. Methods

### 2.1. Participants

We randomly assigned 70 primary schools located in various districts of Hong Kong into either the intervention (*n* = 35) or control (*n* = 35) condition. School officials were contacted by phone, and information on the assigned program was explained. In total, 16 schools in the intervention condition and 23 schools in the control condition agreed to participate. 

These schools distributed invitation letters to all the parents of Primary 4 to 6 students, and 374 parents responded to the invitation to voluntarily participate in the program. Participants were eligible for a lucky draw of five supermarket coupons of 500 Hong Kong dollars (equivalent to 65 US dollars) to compensate for their time and effort. Among the 374 parents, 2 reported that their children had never played video games and 10 reported that their children, on average, spent 0 min playing video games per week. After excluding these parents of non-gamers, the final sample comprised 163 parents in the intervention condition (77% women; age range = 30–62, *M* = 42.70, *SD* = 5.79) and 199 in the control condition (83% women; age range = 29–65, *M* = 41.82, *SD* = 5.80). Approximately 10% of participants in the intervention condition and approximately 11% of participants in the control condition did not complete the final survey. The participants who dropped out and those who completed the entire study did not differ in the reports of their children’s criterion variables measured at baseline, including video gaming time, severity of gaming disorder, and exposure to violent video games (*p*s > 0.55).

### 2.2. Procedures

All of the training sessions were held in an activity room in the school where the participants were recruited. Prior to training, all parents who agreed to participate were given information on the study. After providing consent, they took part in a baseline survey in which self-report questionnaires were administered before the training session began (Time 1). Then, parents in the intervention condition received 4-h training on gaming disorder prevention for children, whereas those in the control condition received 4-h training on effective learning for children. A week (Time 2) and 3 months (Time 3) after attending the training, parents from both conditions were contacted again to complete post-training surveys. The same questionnaires were administered through a telephone interview in which the participants answered all of the questions at home. Debriefing was conducted at the end of the entire study. Those who finished the surveys of all three time points were entered into a lucky draw. 

To match the participants’ data collected from various time points, the participants’ names were used for data matching. A research assistant replaced each of their names with a unique participant number upon completion of the data matching task. The file containing the participants’ names and contact numbers was password-protected, and then was deleted upon completion of the data matching. This study’s protocol had received prior approval from the human research ethics committee of the University of Hong Kong (ethical approval number: EA1508023).

### 2.3. Training

The intervention program comprised three modules: parental monitoring, parental care, and psychoeducation. In the parental monitoring module, parents were shown how to keep track of the time their children spent on gaming (e.g., by using in-game applications) and the content they were exposed to (e.g., by researching on game-rating websites). Parents were also reminded of the importance of monitoring of children’s social interactions in games. To facilitate the practice of such monitoring, a list of questions (e.g., Are you a member of any guilds?) were provided so that parents could use them to initiate conversations on in-game friendships with their children. More importantly, parents were guided to design a token system [43] that would encourage goal setting and record keeping simultaneously. They could use the token system to have open discussions about monitoring activities with their children, which should minimize children’s perceptions of over-controlling parenting.

In the parental care module, there were three elements of focus: (1) expressions of warmth, (2) responsiveness to needs, and (3) cultivation of shared positive emotions. For the first element, parents familiarized with how to express warmth to their children through means such as adopting more open and less closed postures [44]. For the second element, parents learned how to detect signs of distress in children and practiced through role-playing how to respond to such signs using skills of active listening [45]. For the last element, parents watched a video on free play to gain ideas for fun activities for the entire family to take part in together.

In the psychoeducation module, parents were offered information on popular types of video games and a list of channels for them to explore more about the games’ content without the need to own and play the games themselves. For example, there were demonstrations on how to test whether a particular game contained violent content by browsing the Entertainment Software Rating Board website. More importantly, in this module, parents were introduced to the antecedents and consequences of gaming. Specifically, they acquired knowledge about children’s motives behind gaming [46] and potential negative effects of play such as gaming disorder and aggression. Parents were guided to reflect on the possible motives which drove their children’s gaming, and then brainstormed alternative activities that could meet the same set of motives. In addition, parents were encouraged to discuss with their children and their spouses the expectations and rewards pertaining to the alternative activities. Instructions in the psychoeducation module were summarized in a video show, which was then followed by group discussions in which parents were given opportunities to ask questions and exchange ideas with both the program instructor and other participants. 

On the other hand, in the control condition, the program had two modules. The focus of the first module was on learning techniques and that of the second module was on learning styles. As in the intervention program, this program also contained the same structure, including skill demonstrations, video shows, and group discussions.

### 2.4. Measures

The three criterion variables which the intervention program targeted to change were children’s gaming time, exposure to violent video games, and symptoms of gaming disorder. Given that children may be too young to provide an accurate assessment of their own gaming time, the degree of violence of the games they play, and symptoms of gaming disorder, all of these variables were assessed by parents. Moreover, parents also reported their demographic information, namely sex, age, marital and educational status, and their child’s sex and age.

To estimate children’s gaming time, parents were asked, “How many days a week does your child play video games?” and “On an average day that your child plays video games, how much time does he or she spend playing?”. Parents’ answers to the two questions were multiplied to approximate the amount of time (in minutes) their child spent playing video games in a week.

To gauge children’s exposure to violent video games, we adapted an assessment item from the protocol of the Growing up with Media survey [47]. Parents were asked to rate, from 1 to 5, how often the video games their children played showed physical fighting, hurting, shooting, and/or killing. Higher scores represent more frequent exposure to violent video games.

To assess children’s severity of gaming disorder, the observer version of the Korean Internet Addiction Scale for Adolescents K-scale [48] was adapted. The scale has 15 four-point Likert items originally designed for assessing Internet addiction. Scale items were modified to fit the context of video gaming. For example, “When not using the Internet, your child cannot concentrate on things and gets anxious” was altered to become “When not playing video games, your child cannot concentrate on things and gets anxious.” The modified scale demonstrated good reliability (Time 1: Cronbach’s α = 0.84; Time 2: Cronbach’s α = 0.90; Time 3: Cronbach’s α = 0.89). After adjusting for reverse scoring, the scale items were summed. A higher sum of scores indicates the endorsement of more symptoms of gaming disorder.

### 2.5. Plan of Data Analysis

We used the SPSS Version 23.0 to perform chi-square and t-test analyses for testing whether the intervention and control groups differed in demographics (i.e., parent’s sex, age, marital and educational status, and child’s sex and age) at baseline levels. Then, we used the general linear model function to conduct mixed-design ANOVAs to test the key hypotheses that the children of parents in the intervention condition would have a greater reduction in the levels of criterion variables, including gaming time, exposure to violent video games, and symptoms of gaming disorder, than children of those in the control condition over time. If the time–condition interaction effect was significant for a criterion variable, post hoc pairwise comparisons with Bonferroni correction would be performed to compare the means across the time points for each condition, respectively.

## 3. Results

The results from the chi-square and t-test analyses showed that the demographic characteristics of participants were comparable between conditions, except: (1) there was a higher percentage of fathers in the intervention condition than in the control condition, *χ*^2^(1, *N* = 362) = 6.70, *p* = 0.01, and (2), the children of parents in the intervention condition were significantly older than the children of those in the control condition, *t*(341) = 2.46, *p* = 0.01. As the inclusion of the child’s age and parent’s sex as covariates in analyses did not change the patterns of findings, we reported the main results without controlling for those variables. Table 1 displays the demographic information for each condition.

Table 2 shows the descriptive statistics for all of the three gaming-related problems for both conditions. As shown in this table, it is noteworthy that participants in the two conditions differed considerably in the baseline ratings of their children’s gaming time, exposure to violent video games, and gaming disorder symptoms (*p*s < 0.02), with higher scores being consistently reported by parents who took part in the intervention condition. As participation was voluntary, such differences suggested that parents who chose to partake in the GOI tended to perceive their children’s gaming problems as more severe.

**Figure 1:**
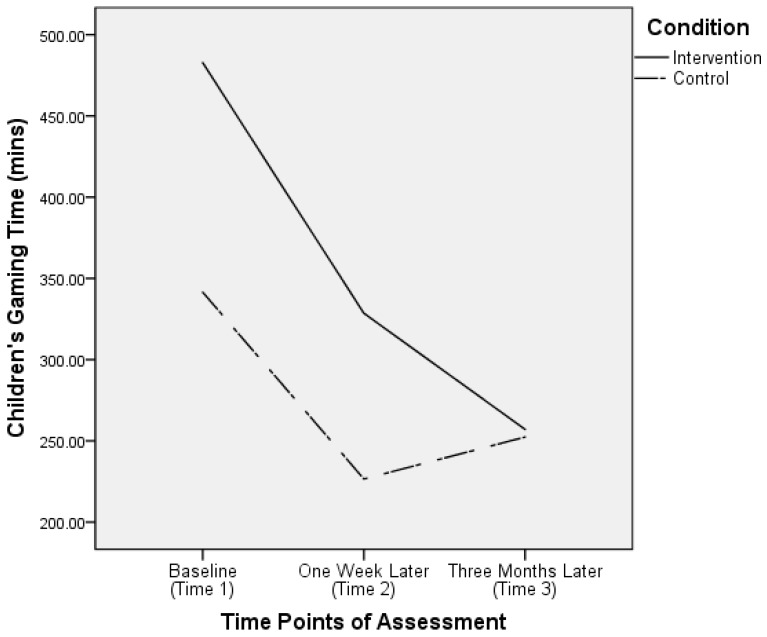
Parent report of children’s gaming time (in minutes) by time and condition.

We performed three sets of mixed-design ANOVAs to test the key hypotheses, one for each of the three criterion variables. For gaming time, the main effect of time (*F*(1.72, 516.22) = 28.43, *p* < 0.001), the main effect of condition (*F*(1, 300) = 4.64, *p* = 0.03) and the time–condition interaction effect (*F*(1.72, 516.22) = 4.86, *p* = 0.01) were all significant. As the results from the Mauchly’s sphericity test were significant (χ^2^(2) = 56.49, *p* < 0.001) and the Greenhouse–Geisser estimate of ε was 0.85, the results regarding the within-participant effects (i.e., main effect of time and time–condition interaction effect) were Huynh–Feldt corrected [49]. Figure 1 depicts the significant interaction effect on gaming time.

We examined the interaction effect using post hoc pairwise comparison analyses. The results revealed that at both Time 1 and Time 2, the average gaming time of the children of parents in the intervention condition was significantly higher than that of the children of parents in the control condition (*p*s < 0.02). Such differences no longer existed at Time 3 (*p* = 0.90). Moreover, in the intervention condition, the average gaming time at Time 1 was significantly higher than that at Time 2 (*p* < 0.001), which in turn was higher than that at Time 3 (*p* = 0.02). However, in the control condition, although the average gaming time at Time 1 was also significantly higher than that at Time 2 (*p* = 0.002), which did not differ from the average gaming time assessed at Time 3 (*p* = 0.79). 

**Figure 2:**
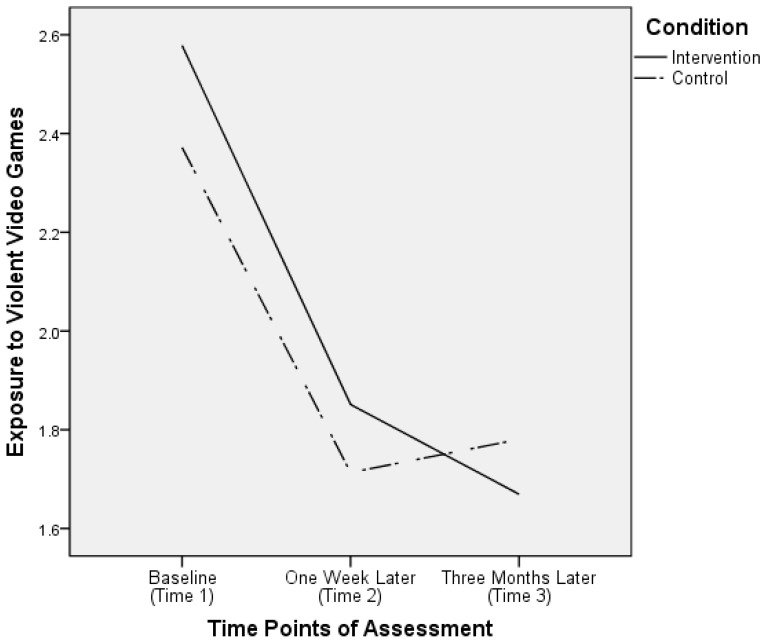
Parent report of children’s exposure to violent video games by time and condition.

For exposure to violent video games, the results showed a significant main effect of time (*F*(2, 566) = 82.90, *p* < 0.001) and a significant time–condition interaction effect (*F*(2, 566) = 3.32, *p* = 0.04). However, the main effect of condition was not significant, *F*(1, 283) = 0.49, *p* = 0.49. No corrections were performed as sphericity was assumed, χ^2^(2) = 4.15, *p* = 0.13. Figure 2 depicts the significant interaction effect on exposure to violent video games. The results from the post hoc pairwise comparison analyses showed that at all three time points, the mean levels of exposure to violent video games did not differ by condition (*p*s > 0.12). Furthermore, in both the intervention and control conditions, the mean level of exposure to violent video games at Time 1 was significantly higher than that at Time 2 (*p* < 0.001 in both conditions), which was not significantly different from that variable measured at Time 3 (intervention condition: *p* = 0.15; control condition: *p* = 1.0). Nevertheless, as shown in Figure 2, there was a reduction in the levels of exposure to violent video games in the intervention condition from Time 2 to Time 3, but an increase in the control condition for the same period.

**Figure 3:**
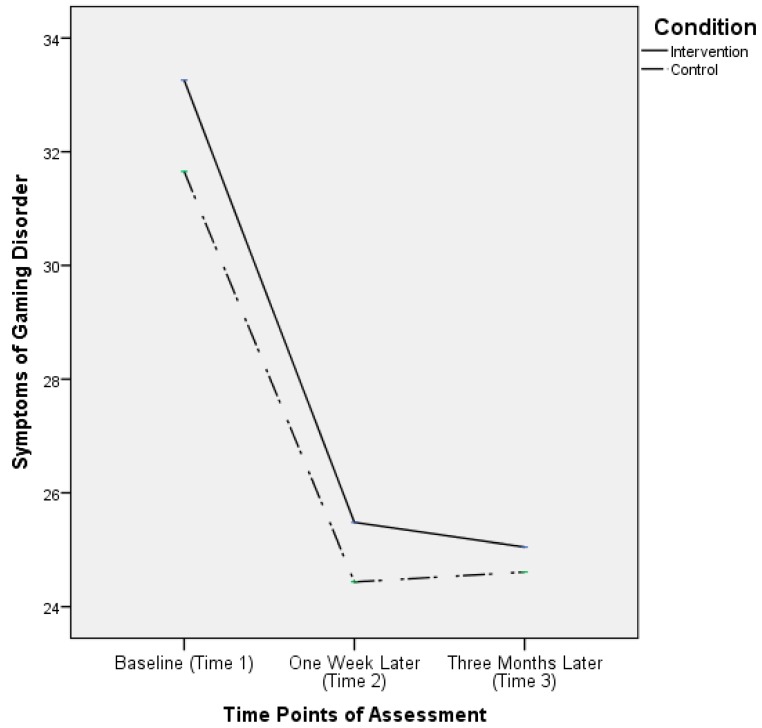
Parent report of children’s gaming disorder symptoms by time and condition.

For symptoms of gaming disorder, there was only a significant main effect of time, *F*(1.93, 520.50) = 281.98, *p* < 0.001. Neither a main effect of condition (*F*(1, 270) = 1.85, *p* = 0.17) nor an interaction effect of time and condition on this criterion variable was significant (*F*(1.93, 520.50) = 1.27, *p* = 0.28). Again, to address the presence of significant Mauchly’s sphericity test results (χ^2^(2) = 13.38, *p* = 0.001) and the Greenhouse–Geisser estimate of ε, 0.95, Huynh–Feldt corrections were applied to the results regarding the within-participant effects. The results are summarized in Figure 3. This figure shows that for the intervention and control conditions, the decrease in symptoms of gaming disorder from Time 1 to Time 2 was significant (*p* < 0.001 in both conditions), and the severity of the symptoms remained the same from Time 2 to Time 3 (*p* = 1.00 in both conditions).

## 4. Discussion

Previous studies have estimated that the prevalence of gaming disorder ranges from 1 to 16% among children [36,50,51], implying that millions of children across the globe suffer from the disorder and its consequences. Yet, there are no theory- and evidence-based gaming disorder prevention programs targeting this population. To fill this gap, we designed a parent-based intervention (i.e., the GOI) based on the self-determination model. The results demonstrated that three months after the GOI, parents reported a significant reduction in their children’s gaming time, levels of exposure to violent video games, and gaming disorder symptoms.

It is noteworthy that similar reductions in the criterion variables are also found for the control condition, although the extent of the changes is generally smaller than those revealed in the intervention condition. Such a lack of group differences may reflect the results of the assessment, rather than the results of the condition. A meta-analysis on the efficacy of therapeutic assessments revealed that when accompanied by feedback delivery, psychological assessments can have moderate effects on treatment processes and outcomes [52]. For the case when psychological assessment was performed alone without feedback delivery, it was still found to improve the intervention goals [53]. In this study, as parents were asked to report on their children’s gaming activities, they might pay more attention to when and what their children played. This could have influenced their monitoring behaviour and contributed to the results as observed.

On the other hand, the trajectories of children’s gaming time and exposure to violent video games differed by condition from Time 2 (one week after intervention) to Time 3 (three months after intervention). In this period, both intervention goals continued to decrease in the intervention condition, but, in contrast, they increased in the control condition. In accordance with the self-determination model, when individuals’ autonomy, competence, and relatedness needs are not met in real life, they will seek to fulfil these needs in their virtual life in games [24]. It is possible that in the three-month period between assessments, parents in the intervention condition did practice the parenting skills that they learned in the program (e.g., parental monitoring and care, reinforcement of alternatives) to satisfy their children’s basic needs. Hence, their children were less distressed and less likely to play video games, especially violent video games. Such improvements were not observed in the children of parents in the control condition because the focus of the control program was on effective learning rather than effective parenting.

Concerning children’s gaming disorder symptoms, there was no significant evidence that the intervention condition was better than the control condition at lowering this intervention target. It might be attributed to the GOI being a universal intervention intended for primary prevention. Participants in this study did not go through any screening of risks their children encountered. Their children might not have any symptoms of gaming disorder at baseline. The developmental process of gaming disorder takes time. 

According to Suissa’s cycle of addiction [54], there are five phases in the development of addiction. First, individuals encounter problems stemming from personal or social vulnerabilities (e.g., poor parenting). Second, they attempt to cope with the problems using substances or displaying certain behaviours (e.g., gaming). Third, they receive temporary emotional rewards (e.g., senses of autonomy, competence, relatedness). Fourth, they experience unpleasant emotions, cognitions, or even problems they encountered in phase one as they are not resolved. Lastly, they choose to either repeat or break away from the cycle. This cycle of addiction can take months to build. In fact, the DSM-5 proposes to assess symptoms of gaming disorder that have arisen over the past twelve months [10]. Hence, in this study, the reason that the significant interaction effects of time and condition on gaming disorder symptoms were not detected might be because it could take longer than 3 months for the symptoms to emerge. Future studies on the efficacy of the GOI should include a longer follow-up period to clarify the present results by evaluating longer-term effects.

### 4.1. Implications and Future Directions

This study shows that the GOI, a parent-based prevention program, is efficacious in changing not only the amount but also the content of children’s gaming activities. These results are consistent with the principles of the ecological systems theory [29] and self-determination model [24], both of which similarly postulate that changes in behaviours can follow changes in the social environment. They also further validate previous findings in which effective parenting was associated with reduced risks for gaming disorder [20,22]. As past studies often used a cross-sectional design, by adopting a randomized controlled trial design, the present study provides more robust evidence of causal relationships between functional parenting and decreased engagement in gaming.

From a practical perspective, it is important to find that a parent-based approach can be applied to changing gaming behaviours. There are two major advantages to using this approach, (1) participants are relatively motivated and (2) service recipients can serve as service providers in future. In comparison with child participants of primary prevention programs, parent participants should be more motivated to engage in early-stage intervention because they are more capable of regulating their thoughts, emotions, and behaviours to achieve long-term goals [55]. Before any gaming-related problems become apparent, child gamers will most likely focus on the immediate effects of playing video games, which can include feelings of excitement, immersion and, more importantly, the short-term fulfilment of basic needs. Unlike children, parents can usually evaluate the long-term costs and benefits of gaming activities and plan proactively to keep problems from occurring, making them excellent agents in preventing problematic gaming in children.

Another advantage of parent training is that parent trainees can later become trainers themselves. Studies have found that pyramidal training for families, which is characterized by the empowerment of one caregiver to educate other family members, increases the overall implementation of intervention within the family [56]. In the GOI, parents are reminded of the importance of and are shown ways to involve and motivate their spouses and other caregivers, such as grandparents and domestic helpers, to engage in the provision of monitoring and care to children. Contrary to individual-based and school-based interventions that have limited sessions, parent-based intervention may have more long-lasting effects because empowered parents and potentially other indirectly trained caregivers can provide ongoing intervention to the children at home [57]. This approach makes a brief universal training highly cost-effective.

Given the efficacy of the GOI, it may be beneficial to expand it from a universal level to selective and indicated levels. Dishion et al. [58] have advocated for the use of a multiple gating strategy in parent-based intervention. “Gating” is metaphorical for screening and risk-based allocation of resources. The premise of the multiple gating strategy is straightforward: more intervention resources should be allocated to the parents of children with greater risks for disorders. At the beginning, all parents receive a brief intervention with elements of psychoeducation. For the parents of children with heightened biological, psychological, or social risks (e.g., special education needs), they and their family are offered individualized assessment and consultation. If subclinical disorders are identified in assessment, services such as group therapy will be provided to families. Applying the multiple gating approach to expanding the GOI, researchers may consider developing selective interventions (e.g., therapeutic assessment) and indicated interventions (e.g., multi-session parenting groups) to systematically lower the prevalence of risks for gaming disorder.

### 4.2. Limitations

This study is not without caveats. To maximize the effects of the GOI, we targeted the parents of upper primary school students. For children in primary schools, what is going on in the family has a great influence on their growth and development. However, as they get older, parental influences may become less significant as peer influences take on more important roles. Studies in the literature have generally supported this observation but have also emphasized that parents still have an impact on children in late adolescence albeit it is relatively small [59]. Under the context of video gaming, this shift in influence is also evident. A qualitative interview revealed that building and maintaining virtual friendships are key motives for MMORPG play [60]. One of the interviewees said, “The reason why I play Final Fantasy is because I’ve made friends there and sometimes I’m not playing but chatting with them. I talk to them more than I do with my family” (p. 153). In addition, recent findings indicate that Asian youngsters with severe symptoms of gaming disorder are more likely to develop problems of anxiety and depression, which in turn lead to a poor health-related quality of life [61]. In this light, we recommend future program evaluations of the GOI to address dynamic developmental and affiliative needs using a greater array of psychosocial indicators, such as health-related quality-of-life and quality of both offline and online peer relations.

In addition, we included an intervention on effective learning in the control condition to test whether any intervention other than the GOI might have the same effects on children. One possible downside of this inclusion is that when parents acquire knowledge and techniques in learning, they may pass that to their children and contribute to their sense of competence. This may have undermined the interaction effects of time and condition on intervention targets. Future studies may consider including a control condition with only assessments to delineate the effects of GOI from those of child development.

## 5. Conclusions

This study aimed at evaluating a new parent-based intervention program, namely the GOI, developed based on ecological systems theory and self-determination theory. The results indicate that parent participants in the intervention condition generally report a reduction in their children’s gaming time, level of exposure to violent video games, and symptoms of gaming disorder three months after taking part in the GOI. Similar changes are found for parent participants who have taken part in the control condition, despite being of a smaller extent compared with that of the changes revealed in the intervention condition. As the participants were only asked to observe these gaming-related phenomena for their children, the absence of differences between the two conditions may reflect the results of assessment rather than condition. Future studies may include more filler items assessing variables unrelated to gaming in order to tackle the problem of demand characteristics. 

## Figures and Tables

**Table 1:** Demographic characteristics of sample by condition.

		Intervention (*n* = 163)	Control (*n* = 199)		
		*M* (*SD*)	*M* (*SD*)	*t*	*p*
Child’s age		10.22 (1.01)	9.97 (.95)	2.32	0.02
Parent’s age		42.75 (5.94)	41.89 (5.71)	1.33	0.18
		Intervention (%)	Control (%)	χ^2^	*p*
Child’s sex	Male	63.2	60.8	0.22	0.64
	Female	36.8	39.2		
Parent’s sex	Male	14.1	6.0	6.70	0.01
	Female	85.9	94.0		
Marital status	Single	0.0	1.0	3.64	0.16
	Married	79.8	86.4		
	Separated/ Divorced/Widowed	10.4	6.5		
	Unknown	9.8	6.0		
Education	Primary/ Lower secondary	34.4	35.2	3.68	0.16
	Upper secondary/ Matriculation/ Associate’s degree	41.7	49.7		
	Bachelor’s degree or above	14.7	9.0		
	Unknown	9.2	6.0		

**Table 2:** Descriptive statistics of three criterion variables by time and condition.

	Intervention (*n* = 163)	Control (*n* = 199)		
	*M* (*SD*)	*M* (*SD*)	*t*	*p*
Gaming time per week (minutes)—Time 1	476.98 (544.81)	343.29 (469.27)	2.58	0.01
Gaming time per week (minutes)—Time 2	316.15 (385.80)	227.91 (319.13)	2.40	0.02
Gaming time per week (minutes)—Time 3	271.90 (294.96)	253.81 (339.75)	0.40	0.69
Exposure to violent video games—Time 1	2.58 (1.06)	2.37 (1.14)	2.41	0.02
Exposure to violent video games—Time 2	1.85 (1.19)	1.71 (1.14)	1.45	0.15
Exposure to violent video games—Time 3	1.67 (0.99)	1.78 (1.15)	−0.59	0.56
Gaming disorder symptoms—Time 1	33.26 (6.15)	31.65 (6.89)	2.79	0.01
Gaming disorder symptoms—Time 2	25.48 (7.88)	24.44 (7.30)	1.58	0.12
Gaming disorder symptoms—Time 3	25.05 (7.00)	24.61 (7.34)	1.22	0.22

## References

[1] NPD Group (2011). Kids and Gaming 2011.

[2] Sung H., Sigerson L., Cheng C. (2017). Social capital accumulation in location-based mobile game playing: A multiple-process approach. Cyberpsychol. Behav. Soc. Netw..

[3] Zach F.J., Tussyadiah I.P., Schegg R., Stangl B. (2017). To catch them all—The (un) intended consequences of Pokémon GO on mobility, consumption, and wellbeing. Information and Communication Technologies in Tourism 2017.

[4] Kuss D.J., Louws J., Wiers R.W. (2012). Online gaming addiction? Motives predict addictive play behavior in massively multiplayer online role-playing games. Cyberpsychol. Behav. Soc. Netw..

[5] Wang H.-Y., Sigerson L., Cheng C. (2019). Digital nativity and information technology addiction: Age cohort versus individual difference approaches. Comput. Hum. Behav..

[6] Decamp W. (2017). Who plays violent video games? An exploratory analysis of predictors of playing violent games. Personal. Individ. Differ..

[7] Calvert S.L., Appelbaum M., Dodge K.A., Graham S., Nagayama Hall G.C., Hamby S., Fasig-Caldwell L.G., Citkowicz M., Galloway D.P., Hedges L.V. (2017). The American Psychological Association Task Force Assessment of Violent Video Games: Science in the Service of Public Interest. Am. Psychol..

[8] Gentile D., Choo H., Liau A., Sim T., Li D., Fung D., Khoo A. (2011). Pathological video game use among youths: A two-year longitudinal study. Pediatrics.

[9] Lemmens J.S., Valkenburg P.M., Peter J. (2011). The Effects of Pathological Gaming on Aggressive Behavior. J. Youth Adolesc..

[10] American Psychiatric Association (2013). Diagnostic and Statistical Manual of Mental Disorders.

[11] Pontes H.M., Griffiths M.D. (2015). Measuring DSM-5 Internet Gaming Disorder: Development and validation of a short psychometric scale. Comput. Hum. Behav..

[12] Sigerson L., Li A.Y.L., Cheung M.W.L., Luk J.W., Cheng C. (2017). Psychometric properties of the Chinese Internet Gaming Disorder Scale. Addict. Behav..

[13] Bargeron A.H., Hormes J.M. (2017). Psychosocial correlates of Internet gaming disorder: Psychopathology, life satisfaction, and impulsivity. Comput. Hum. Behav..

[14] Peng W., Liu M. (2010). Online gaming dependency: A preliminary study in China. Cyberpsychol. Behav. Soc. Netw..

[15] Pontes H.M., Griffiths M.D. (2016). Portuguese validation of the Internet Gaming Disorder Scale-Short-Form. Cyberpsychol. Behav. Soc. Netw..

[16] Sigerson L., Li A.Y.L., Cheung M.W.L., Cheng C. (2017). Examining common information technology addictions and their relationships with non-technology-related addictions. Comput. Hum. Behav..

[17] Wang C.-W., Ho R.T.H., Chan C.L.W., Tse S. (2015). Exploring personality characteristics of Chinese adolescents with Internet-related addictive behaviors: Trait differences for gaming addiction and social networking addiction. Addict. Behav..

[18] Yen J.-Y., Liu T.-L., Wang P.-W., Chen C.-S., Yen C.-F., Ko C.-H. (2017). Association between Internet gaming disorder and adult attention deficit and hyperactivity disorder and their correlates: Impulsivity and hostility. Addict. Behav..

[19] Li A.Y., Lo B.C.Y., Cheng C. (2018). It is the family context that matters: Concurrent and predictive effects of aspects of parent-child interaction on video gaming-related problems. Cyberpsychol. Behav. Soc. Netw..

[20] Siomos K., Floros G., Fisoun V., Evaggelia D., Farkonas N., Sergentani E., Lamprou M., Geroukalis D. (2012). Evolution of Internet addiction in Greek adolescent students over a two-year period: The impact of parental bonding. Eur. Child Adolesc. Psychiatry.

[21] Yao M.Z., He J., Ko D.M., Pang K. (2014). The influence of personality, parental behaviors, and self-esteem on Internet addiction: A study of Chinese college students. Cyberpsychol. Behav. Soc. Netw..

[22] Zhu J., Zhang W., Yu C., Bao Z. (2015). Early adolescent Internet game addiction in context: How parents, school, and peers impact youth. Comput. Hum. Behav..

[23] Bronfenbrenner U., Vasta R. (1992). Ecological systems theory. Six Theories of Child Development: Revised Formulations and Current Issues.

[24] Ryan R.M., Deci E.L. (2000). Self-determination theory and the facilitation of intrinsic motivation, social development, and well-being. Am. Psychol..

[25] Chen Y., Goh K., Razak M.A. (2012). Development of a Web-Based Tailored Intervention for Excessive Gaming.

[26] Toker S., Baturay M.H. (2016). Antecedents and consequences of game addiction. Comput. Hum. Behav..

[27] De Leeuw J.R.J., de Bruijn M., de Weert-van Oene G.H., Schrijvers A.J.P. (2010). Internet and game behaviour at a secondary school and a newly developed health promotion programme: A prospective study. BMC Public Health.

[28] Cheng C., Cheung M.W.L., Wang H.-Y. (2018). Multinational comparison of internet gaming disorder and psychosocial problems versus well-being: Meta-analysis of 20 countries. Comput. Hum. Behav..

[29] Bronfenbrenner U. (1979). Contexts of child rearing: Problems and prospects. Am. Psychol..

[30] Ding Q., Li D., Zhou Y., Dong H., Luo J. (2017). Perceived parental monitoring and adolescent internet addiction: A moderated mediation model. Addict. Behav..

[31] Przybylski A.K., Rigby C.S., Ryan R.M. (2010). A motivational model of video game engagement. Rev. Gen. Psychol..

[32] Lin C.-H., Lin S.-L., Wu C.-P. (2009). The effects of parental monitoring and leisure boredom on adolescents’ Internet addiction. Adolescence.

[33] Caldwell L.L., Darling N., Payne L.L., Dowdy B. (1999). “Why are you bored?”: An examination of psychological and social control causes of boredom among adolescents. J. Leis. Res..

[34] Parker J.S., Benson M.J. (2004). Parent-adolescent relations and adolescent functioning: Self-esteem, substance abuse, and delinquency. Adolescence.

[35] Grolnick W.S., Farkas M., Bornstein M.H. (2002). Parenting and the development of children’s self-regulation. Handbook of Parenting: Volume 5. Practical Issues in Parenting.

[36] Griffiths M.D., Kuss D.J., Pontes H.M. (2016). A brief overview of Internet gaming disorder and its treatment. Aust. Clin. Psychol..

[37] Raftery J.N., Grolnick W.S., Flamm E.S., Christenson S.L., Reschly A.L., Wylie C. (2012). Families as facilitators of student engagement: Toward a home-school partnership model. Handbook of Research on Student Engagement.

[38] Eggen B., Feijs L., Peters P. Children’s Participation in the Design of Physical and on Screen-Intervention Strategies to Prevent Excessive Game Play. Proceedings of the 6th Asian Design Conference.

[39] American Psychological Association (2014). Guidelines for prevention in psychology. Am. Psychol..

[40] Kuss D.J., Griffiths M.D. (2012). Online gaming addiction in children and adolescents: A review of empirical research. J. Behav. Addict..

[41] Yee N. (2006). Motivations for play in online games. Cyberpsychol. Behav..

[42] Bailey K., West R., Anderson C.A. (2010). A negative association between video game experience and proactive cognitive control. Psychophysiology.

[43] Kapalka G. (2007). Parenting Your Out-of-Control Child: An Effective, Easy-to-Use Program for Teaching Self-Control.

[44] Borg J. (2008). Body Language: 7 Easy Lessons to Master the Silent Language.

[45] Gordon T. (2000). Parent Effectiveness Training: The Proven Program for Raising Responsible Children.

[46] Olson C.K. (2010). Children’s motivations for video game play in the context of normal development. Rev. Gen. Psychol..

[47] Ybarra M.L., Diener-West M., Markow D., Leaf P.J., Hamburger M., Boxer P. (2008). Linkages between Internet and other media violence with seriously violent behavior by youth. Pediatrics.

[48] National Information Society Agency (2011). Third standardization of Korean Internet Addiction Proneness Scale. NIA Research Report.

[49] Girden E.R. (1992). ANOVA: Repeated Measures.

[50] Festl R., Scharkow M., Quandt T. (2013). Problematic computer game use among adolescents, younger and older adults. Addiction.

[51] Wang C.-W., Chan C.L.W., Mak K.-K., Ho S.-Y., Wong P.W.C., Ho R.T.H. (2014). Prevalence and Correlates of Video and Internet Gaming Addiction among Hong Kong Adolescents: A Pilot Study. Sci. World J..

[52] Poston J.M., Hanson W.E. (2010). Meta-analysis of psychological assessment as a therapeutic intervention. Psychol. Assess..

[53] Young A.S., Meers M.R., Vesco A.T., Seidenfeld A.M., Arnold L.E., Fristad M.A. (2016). Predicting therapeutic effects of psychodiagnostic assessment among children and adolescents participating in randomized controlled trials. J. Clin. Child Adolesc. Psychol..

[54] Suissa A.J. (2014). Cyberaddictions: Toward a psychosocial perspective. Addict. Behav..

[55] Steinberg L. (2005). Cognitive and affective development in adolescence. Trends Cogn. Sci..

[56] Kuhn S.A.C., Lerman D.C., Vorndran C.M. (2003). Pyramidal training for families of children with problem behavior. J. Appl. Behav. Anal..

[57] Dell S. (1974). Training parents in behavior modification: A review. Psychol. Bull..

[58] Dishion T.J., Kavanagh K., Kiesner J., Ashery R.S., Robertson E.B., Kumpfer K.L. (1998). Prevention of early adolescent substance abuse among high-risk youth: A multiple gating approach to parent intervention. Drug Abuse Prevention through Family Interventions.

[59] Wood M.D., Read J.P., Mitchell R.E., Brand N.H. (2004). Do parents still matter? Parent and peer influences on alcohol involvement among recent high school graduates. Psychol. Addict. Behav..

[60] Beranuy M., Carbonell X., Griffiths M.D. (2013). A qualitative analysis of online gaming addicts in treatment. Int. J. Ment. Health Addict..

[61] Tran B.X., Huong L.T., Hinh N.D., Nguyen L.H., Le B.N., Nong V.M., Thuc V.T.M., Tho T.D., Latkin C., Zhang M.W. (2017). A study on the influence of internet addiction and online interpersonal influences on health-related quality of life in young Vietnamese. BMC Public Health.

